# What the Microscope Reveals in the Decay of the Teeth

**Published:** 1874-07

**Authors:** Geo. B. Harriman

**Affiliations:** Boston, Mass.


### WHAT THE MICROSCOPE REVEALS IN THE DECAY OF THE TEETH.

BY GEO. B. HARRIMAN, M. D., D. D. S., BOSTON, MASS.

Writers in our dental journals advocate various theories concerning the decay of the teeth. One is, that the mucous secretions of the mouth being acid produce this decay. Another is the theory of Drs. Leber and Rottenstein, viz: "The action of acids and the rapid development of parasitic plant, the Leptothrix buccalis."

This they pronounce the great destroyer of the natural teeth.

An examination, both microscopical and chemical, of the mucous secretions may aid in arriving at the true cause of this common decay and thus benefit the dental profession.

The healthy mucous exudations, when examined under the microscope, are strongly allied to the fibrin in the blood, and it is extremely difficult to detect any difference without frequent and careful microscopical examinations by a skilled and practical operator. Such examinations will discover in these secretions an albuminous substance resembling the white of an egg and is very similar to gelatine.

This examination will reveal numerous flattened epithelial scales with granular bodies and few oil globules. When this examination is made with 1-50 or 1-75 object glass very minute bioplasms* or spores are readily detected. In certain diseased conditions of the mucous membrane and dental organs, diversified forms of bioplasms are numerous. In Lehmann's "Physiological Chemistry," we find the chemical analysis of mucous as follows: Carbon 52, hydrogen 6.97 nitrogen 12.82, oxygen 28.11,and white ash 4.04, which contains some alkaline carbonates, together with a tolerably large quantity of phosphate of lime." Lehmann further observes, "mucous is very rich in alkali."

In my examination of fresh mucous exudations from the mouths of upwards of sixty patients, I have entirely failed to discover anything of an acid character, except in diseased conditions of the system, and where the food spores and broken down epithelial scales and the saliva are allowed to remain for several hours between and around the teeth. In such cases, I have discovered a slight acid reaction.

The result of these repeated examinations, microscopical and chemical, show healthy fresh mucous to contain fibrin and albumen of an alkaline character, but no acids. The test paper discloses saliva, in its normal condition to be alkaline.

*Beale on "Disease Germs."

It may be profitable now to examine the "Parasitic plant," Leptothrix buccalis. The microscopist engaged in this examination discovers the Leptothrix buccalis in the uncalcified and disintegrated portions of the enamel and dentine, and in the dark fissures, many of which are apparent to the naked eye, but other cases only by the aid of the microscope.

In the interstices, we find these Leptothrix buccalis, which resemble mould on bread or meat. But though of a different hue, it is in reality the same thing, having the same confervoid growth and may be properly called bioplasm,* Beale applying this term to any living growing granule. The highest powers employed by Drs. Leber and Rottenstein were only two hundred and fifty diameters.

In using the 1-50 and 1-75 objective with a magnifying power of from five to ten thousand diameters in these examinations, I find much more than this simple confervoid growth, the Leptothrix buccalis, which I prefer to call spores of bioplasm.

We also discover bacteria, amæbœ, monads, vibriones and countless numbers of small spores, which are constantly undergoing changes, some of which are not more than the one hundred and fifty thousandth of an inch in diameter. There can be no doubt that they work to the injury of the teeth.

It is a pertinent inference from the above premises and what examinations will sustain, that in the anomalies of the dental organs will be discovered food, broken down epithelial scales, spores and mucous, creating acid which adheres in the fissures and around the teeth for some hours, thus helping the disintegration of the tooth substance by softening the phosphate of lime and changing the fibrous portion (so-called tubes) into spores.

We also discover other causes aiding in the destruction of the natural teeth, to wit: alkalies. A strong alkaline solution will act with about as much power in destroying the teeth as mineral acids. Admit that, at times, we find both acid and alkaline conditions of the mouth, are these, with the spores which can be found in every individual mouth, sufficient to

*"Disease Germs."

destroy well developed normal teeth? We think not. We are to go back to the development, when the salts of lime are being deposited, before the tooth is erupted, to discover the wise provision of Nature, with proper nutritious substances, to produce well developed and healthy normal teeth.

From an examination of over one hundred patients, from the age of seven years and upwards, both with the naked eye and low magnifying power of the microscope, it is my opinion that I have been unable to detect a single perfectly normal tooth. In many instances, to the naked vision, the teeth presented the appearance of health and strength, but, when examined with the microscope, their structure was found irregular and defective. In the case of some, five and six dark fissures could readily be seen, in others, one and sometimes more abnormal sulci were detected, the great proportion of the anomalies being, without doubt, due to abnormal development; the want of the proper material, the salts of lime for the production of healthy teeth.

One word more, and I will close; I wish to impress upon the mind of every microscopist in the dental profession, the great importance of using higher power objectives in these examinations than those used by a majority of the profession. They will find their investigations much more interesting, and will find many objects that can not be seen by the use of low powers.

